# Research on the relationship between parents' media literacy and preschoolers' learning quality: the mediating role of preschoolers' electronic device use

**DOI:** 10.3389/fpsyg.2026.1792526

**Published:** 2026-06-16

**Authors:** Zhenglin Gu, Shanshan Zhang, Yang Wang, Hui Du

**Affiliations:** 1 Faculty of Education, Yunnan Normal University, Kunming, China; 2 Department of Physical Education, Kunming Medical University, Kunming, China; 3 School of Physical Education, Yunnan Normal University, Kunming, China

**Keywords:** mediating role, family media education, parents' media literacy, young children's electronic product usage, young children's learning quality

## Abstract

**Introduction:**

In the digital era, electronic products have become deeply embedded in the daily life and learning of young children. Parents' own media literacy is related to the appropriateness of children's exposure to electronic devices. Fostering young children's learning quality requires parents to cultivate a healthy family media education environment. This study attempts to construct a mediation model to examine how parents' media literacy is linked to young children's electronic product usage and learning quality, and aims to explore the interaction mechanisms and influence paths among these three variables.

**Methods:**

A total of 331 questionnaires were collected through online surveys. Descriptive statistics, correlation analysis, and regression analysis were employed to examine the relationship between parents' media literacy and young children's learning quality, and the mediating role of young children's electronic product usage between the two was also analyzed.

**Results:**

The results revealed that parental media literacy was significantly positively correlated with young children's digital device use. Meanwhile, parental media literacy was also significantly positively associated with young children's learning quality. When digital device use was introduced as a mediating variable, both parental media literacy and young children's digital device use showed significant correlations with children's learning quality. Digital device use exerted a partial mediating effect between parental media literacy and young children's learning quality, with an indirect effect of 0.080.

**Discussion:**

Based on the above findings, the researchers propose targeted educational suggestions: enhancing parents' media literacy to foster a positive family media environment; cultivating appropriate electronic product usage habits in young children to exert the positive effects of such products; and attaching great importance to the cultivation of learning quality to lay a solid foundation for young children's lifelong development.

## 1 Problem formulation

With the rapid advancement of science and technology and the continuous evolution of social demands, the global trend of educational development is undergoing transformation. The Blue Book on Adolescents: Report on Internet Usage among Minors in China (2023) indicates that the number of minor internet users in China has reached 183 million, with the age of first internet access among minors trending younger—even extending to preschool children (Qu, 2024). Compared with students of other age groups, young children have unique characteristics as a group of electronic product users: they possess limited life experience and knowledge reserves. Whether their use of electronic products is scientific largely depends on the level of parents' media literacy. Families are often the starting point for young children's exposure to electronic products; parental intervention and guidance help young children develop habits of using electronic products rationally. The quality of parents' media literacy directly affects young children, and this impact is enduring.

*Guidelines for the Learning and Development of Children Aged 3–6* points out that solely focusing on the imparting of knowledge and skills while neglecting the cultivation of learning quality is not conducive to young children's long-term progress; such an approach is short-sighted and detrimental (Ministry of Education of the People's Republic of China, 2012). In recent years, educational policy documents issued by the national and local governments have repeatedly emphasized the cultivation of young children's learning quality, which indirectly reflects the important role of learning quality in young children's development and also indicates that high-quality preschool education facilitates young children's social development. While electronic products bring convenience to life, they exert a profound impact on young children's learning quality, primarily due to young children's poor self-control ability.

To sum up, with the advancement of information technology, young children's exposure to electronic products has shown a normalized tendency. In response to the complex media environment, attaching importance to the cultivation of young children's learning quality and their scientific use of electronic products has become a key issue in current educational development. This study attempts to explore the relationship between parents' media literacy and young children's learning quality, and further examine whether young children's use of electronic products plays a mediating role between the two. It is expected to put forward practical educational suggestions for creating a positive parental media education environment and enhancing young children's learning quality.

### 1.1 Parents' media literacy

The concept of media literacy can be traced back to Britain in the 1930s, when F. R. Leavis and Denise Thompson first proposed media literacy. Liu (2015)), a Chinese scholar, provided the first detailed definition of parental media literacy, arguing that media literacy in the broad sense is not entirely equivalent to parental media literacy. Parental media literacy involves more dimensions than general adult media literacy: in addition to the basic media literacy required of citizens, it also includes the unique educational literacy possessed by parents. Using concept mapping, Lee and Kwon (2024)) conducted a study on parents of preschool children and identified six dimensions of parental media literacy: management of media use rules, screening of media information, media selection and evaluation, educational application of media content, support for media use, and online problem-solving. Regarding research on the current status of parental media literacy, Chen and Li (2015)) found that parents of young children demonstrate relatively strong abilities in accessing media information, yet require further improvement in media knowledge and media judgment skills. Qu (2020)) showed that mothers of young children exhibit satisfactory basic media literacy and media education awareness, but tend to adopt lenient management over their children's media use and lack appropriate educational guidance. According to the Interactional Theory of Childhood Problematic Media Use, parents' behavioral habits and values related to media use are among the proximal factors contributing to problematic media use in children (Domoff et al., 2020). In summary, a review of existing studies reveals that the connotation of parental media literacy differs from that of general citizen media literacy. Beyond basic media literacy, it also includes the specific media education literacy demonstrated by parents in family education. Current literature mentions parental media literacy of young children in a fragmented manner, indicating that further exploration of parental media literacy is still warranted.

### 1.2 Research on learning quality

Regarding the definition of learning qualities, Wang et al. (2010)) regarded learning qualities as an important component of children's school readiness, referring to the factors that support children's academic achievement, with a focus on the definitional scope of children's learning qualities. The State of Washington in the United States defines learning qualities as how children acquire knowledge and skills, rather than which specific skills they master. In 2017, New Zealand revised its national early childhood education curriculum framework, in which it clearly specified the components of learning qualities. These include courage, curiosity, trust, playfulness, persistence, confidence, responsibility, as well as reciprocity, creativity, imagination, and resilience (Ministry of Education, New Zealand, 2017). The Guidelines for Early Childhood Learning and Development (Ages 3–6) particularly emphasizes the importance of fostering children's learning qualities. It calls for respecting and protecting children's curiosity and desire to explore, and guiding them to develop positive learning qualities such as initiative, concentration, courage in the face of difficulty, willingness to inquire and experiment, and joy in imagination and creation (Ministry of Education of the People's Republic of China, 2012). Yan (2009)) divided learning qualities into five dimensions: curiosity and interest, initiative, persistence and attention, creativity and invention, and reflection and interpretation. In summary, in both educational research and practice, learning qualities are regarded as a key dimension of early childhood development, whether at the macro level of educational policy or the micro level of academic research. They are characterized by a focus on non-specific knowledge and skills, reflecting the dynamic process through which children acquire and apply knowledge, as well as the positive psychological states and sustainable behavioral patterns demonstrated during activities.

Regarding the characteristics of learning quality, Chinese scholars Gong et al. (2024)) found that the longer parents live with their young children, the higher the quality of the children's habit formation; parent-child interaction plays a particularly effective role in promoting young children's enthusiasm for exploration, autonomy, and perseverance. Li (2023)) discovered that teacher-child interaction quality can significantly affect the development of young children's learning quality, and young children's curiosity and initiative are significantly correlated with teachers' behavior of adopting students' ideas during teacher-child interaction. To sum up, the development of young children's learning quality is influenced by the interplay of multiple factors, including young children's individual differences, the family ecosystem, and the environment of preschool educational institutions. As core agents in the educational process, parents and teachers play a positive role in promoting the development of young children's learning quality. Therefore, to promote the development of young children's learning quality, it is necessary to explore the interaction mechanisms among these influencing factors.

### 1.3 Young children's use of electronic products

In recent years, with the prevalence of electronic devices in young children's daily lives, researchers have paid increasing attention to issues surrounding young children's exposure to and use of electronic products. Regarding the duration of young children's electronic device use, a survey by Luo and Liu (2023)) showed that 3% of young children used electronic products once or twice a week, 13% used them every two days, and a striking 74% used them daily. In a study of 514 five-year-old children and their mothers in South Korea, Moon and Shin (2025)) found that young children averaged 1.59 h of daily digital device use. Concerning the content of young children's electronic product use, research by Li and Liu (2022)) indicated that young children's screen-based activities primarily include watching television, listening to children's songs and stories via early-learning devices and storytellers, playing with smartphones, and viewing educational videos—ranked by duration in descending order. Existing research shows that the content of young children's electronic device use is diverse but dominated by entertainment. Parents and teachers should therefore guide children's use scientifically to achieve an organic integration of entertainment and educational value. On the impacts of young children's electronic device use, a study by Wang et al. (2021)) on sleep quality among children aged 3–6 revealed that prolonged use is strongly associated with sleep problems. Xiong et al. (2022)) found that television viewing displaces time that would otherwise be allocated to other activities—many of which promote executive function development in preschoolers. By reducing time spent on such activities, television exposure exerts a negative effect on preschoolers' executive function. In a large-scale German cohort study led by Poulain et al. (2019)), researchers identified a negative correlation between family socioeconomic status (SES) and children's media misuse: higher family SES was associated with lower tendencies toward problematic media use among children (Poulain et al., 2019). Thus, there is an urgent need for parents and educators to shift traditional mindsets regarding young children's electronic device use. By optimizing usage duration, selecting age-appropriate content, and improving interactive engagement, adults can lay a solid foundation for children's holistic development and their future adaptation to the digital society.

Based on the studies of the above scholars, most of the current studies focus on the effects of family environment characteristics and parental parenting styles on young children's learning quality. However, there is still a lack of research on the relationship between parental media literacy and young children's learning quality. Firstly, existing studies have not yet fully confirmed the relationship between parental media literacy and the development of young children's learning quality, and relevant research needs to be further strengthened. Secondly, in the context of digitalization, the interaction mechanism among parental media literacy level, young children's use of electronic products, and the development of young children's learning quality has not been fully explored. Based on previous studies, this study explores the current situation of parental media literacy, young children's use of electronic products, and young children's learning quality; sets young children's use of electronic products as a mediating variable to explore the relationship between parental media literacy and young children's learning quality, further explores whether young children's use of electronic products plays a mediating role between the two, and puts forward suggestions for creating a good parental media parenting atmosphere and promoting the improvement of young children's learning quality.

## 2 Research methods

### 2.1 Research hypotheses

Bronfenbrenner (1981))'s “Ecological Systems Theory” explains that individual development occurs within a complex network of multiple systems, ranging from immediate environments (e.g., family) to indirect ones (e.g., culture). Among these, the microsystem serves as the core of the human ecological development system and exerts a direct impact on individuals' growth. Families and kindergartens are the primary activity environments for preschoolers, where the use of electronic devices mostly takes place. In kindergartens, electronic device usage is mostly organized by teachers, making it difficult to determine preschoolers' true intentions and thus failing to accurately grasp their actual needs and tendencies regarding such devices. In contrast, preschoolers are relatively free to decide on the use of electronic devices in the family environment, and this process can influence their learning qualities.

**Figure 1:**
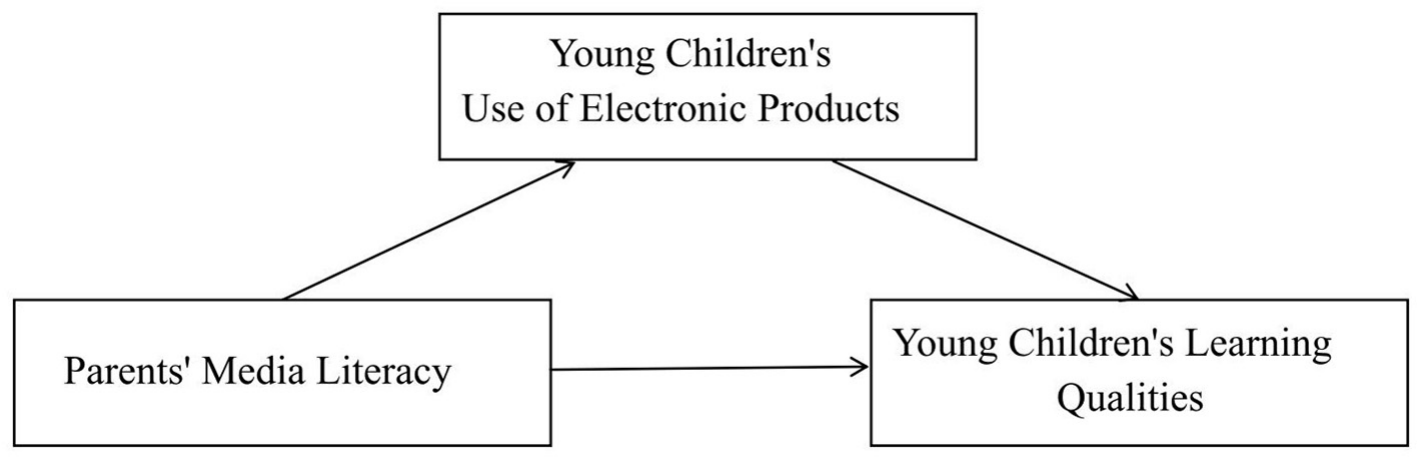
The conceptual mediation model.

Feuerstein's (R. Feuerstein) Theory of Mediated Learning Experience (MLE) suggests that there are two modes of development of children's cognitive structure: one is learning through direct interaction with the environment, and the other is learning through mediation (Liu, 1988). Based on this, the present study takes parents and preschoolers who participated in the questionnaire survey as the research objects, conducts the research from the perspective of the Theory of Mediated Learning Experience, and attempts to construct a mediating model where parents' media literacy influences preschoolers' use of electronic devices and their learning qualities. It further explores the interaction mechanisms and influence paths among the three variables, with the aim of improving parents' media literacy and enhancing preschoolers' learning qualities (see Figure 1).

The research hypotheses are as follows: (1) Parents' media literacy is significantly associated with preschoolers' learning qualities; (2) Preschoolers' use of electronic devices is significantly related to parents' media literacy; (3) Preschoolers' use of electronic devices shows a significant correlation with their learning qualities; (4) Preschoolers' use of electronic devices plays a mediating role between parents' media literacy and preschoolers' learning qualities.

### 2.2 Research objects

This study adopted convenience sampling and selected parents of children aged 3–6 years from several kindergartens in Yunnan Province as research participants between March and April 2025. Questionnaire links and QR codes were distributed by kindergarten teachers via parent WeChat groups to invite eligible parents to participate voluntarily. Inclusion criteria were as follows: children aged 3–6 years; parents serving as the actual primary caregivers; and informed consent to participate voluntarily. Questionnaire distribution and collection were conducted online through the Wenjuanxing platform. A total of 350 questionnaires were distributed, and 341 were returned. After excluding 10 invalid questionnaires (exclusion criteria: incomplete responses or obvious logical errors), 331 valid datasets were obtained, representing a valid response rate of 94.57%. The 331 valid samples were from Yunnan Province, China. In terms of regional characteristics, Yunnan's per capita GDP is approximately 70% of the national average, its urbanization rate is about 15% points lower than that of eastern provinces, the ethnic minority population accounts for more than 33%, and it has 25 border counties. These indicators are highly consistent with the overall characteristics of western China, such as Sichuan, Guizhou, and Gansu provinces. Therefore, although convenience sampling was used and the sample was concentrated in Yunnan Province, its features in economic development, geographical location, cultural diversity, and digital access appear to be broadly consistent with the general situation of less developed western regions of China, suggesting that the research conclusions may be generalizable to similar regions. The basic characteristics of the sample are analyzed below.

As shown in Table 1, among the valid samples collected in this survey, 144 children were boys, accounting for 43.5% of the total, and 187 were girls, accounting for 56.5% of the total. The gender distribution was relatively balanced, with a slightly higher proportion of girls. In terms of age, 137 children aged 3–4 years accounted for 41.4% of the total; 91 children aged 4–5 years accounted for 27.5%; and 103 children aged 5–6 years accounted for 31.1%. The 3–4 age group constituted the largest proportion in the sample. Regarding parental education level, 90 parents had a high school education or below, accounting for 27.2%; 190 held a junior college or bachelor's degree, accounting for 57.4%; and 51 held a postgraduate degree or above, accounting for 15.4%. Overall, the participating parents were relatively well-educated, with 72.8% having received higher education (bachelor's degree or above). In terms of household registration type, 280 families were from urban areas, accounting for 84.6% of the total, while 51 families were from rural areas, accounting for 15.4%.

**Table 1:** Basic information of the survey sample.

Item	Category	Sample size *(N)*	Percentage (%)
Child gender	Male	144	43.5%
	Female	187	56.5%
Child age	3–4 years old	137	41.4%
	4–5 years old	91	27.5%
	5–6 years old	103	31.1%
Parental education level	High school or below	90	27.2%
	Junior college or bachelor's degree	190	57.4%
	Postgraduate or above	51	15.4%
Family location	Urban	280	84.6%
	Rural	51	15.4%

### 2.3 Research instruments

This study distributed an online questionnaire to parents of children aged 3–6 years. Based on previous research, the Questionnaire on the Relationship Between Parental Media Literacy and Young Children's Learning Quality was developed. The questionnaire consists of four parts: (1) basic demographic information of respondents; (2) parental media literacy; (3) young children's electronic device use; (4) young children's learning quality. The questionnaire contains 69 items in total. The first 5 items cover basic information, and items 6 to 69 are scale items measured using a 5-point Likert scale.

The Parental Media Literacy Scale was adopted from the Questionnaire on Media Literacy of Parents of Children Aged 3–6 developed by Zou (2023)), which includes six dimensions: media information access, media information analysis, media information participation, media education methods, media education beliefs, and media-related parent-child interaction ability. In this study, the Cronbach's α coefficient for parental media literacy was 0.952, indicating good reliability.

The Young Children's Electronic Device Use Scale was adopted from the Questionnaire on Young Children's Electronic Device Use developed by Sun (2021)), with six dimensions: time control, frequency control, emotional reactions when stopping use, sociality, desire to use electronic devices, and physical impacts. Note that this scale uses reverse scoring; therefore, raw data were recoded so that higher scores indicate more severe problems in children's electronic device use. The Cronbach's α coefficient for this scale was 0.964, indicating good reliability.

The Young Children's Learning Quality Scale was adopted from Sun (2021)), including five dimensions: curiosity and interest, initiative, persistence and attention, reflection and interpretation, and creativity and invention. The Cronbach's α coefficient for this scale was 0.963, indicating good reliability. Considering the overall reliability level, Table 2 shows that the overall Cronbach's α coefficient reached 0.976, indicating excellent overall reliability of the questionnaire.

**Table 2:** Reliability statistics of the questionnaires.

Variable	Cronbach's α	Number of items
Parents' media literacy	0.952	19
Young children's use of electronic products	0.964	22
Young children's learning qualities	0.963	23
Total (All items combined)	0.976	64

The three scales used in this study were adopted from existing research, and their structural validity has been verified by exploratory factor analysis (EFA) in the original studies. The parental media literacy scale was adopted from Zou, 2023. Exploratory factor analysis was performed on its 20 items, yielding a KMO value of 0.907 and a significant Bartlett's test of sphericity (*p* < 0.001). The correlation coefficients between each dimension and the total score ranged from 0.71 to 0.89, indicating good structural validity. The original study identified four dimensions—basic media literacy, media education beliefs, media education methods, and media-related parent-child interaction ability—based on eigenvalues >1 and the theoretical framework. The young children's electronic device use scale and the young children's learning quality scale were adopted from Sun Mengzhu's master's thesis. In the original study, exploratory factor analysis was conducted separately for both scales. The results showed that the KMO value of the electronic device use scale was 0.853 with a significant Bartlett's test (*p* < 0.001), and the correlation coefficients between each dimension and the total score ranged from 0.56 to 0.85. For the learning quality scale, the KMO value was 0.877 with a significant Bartlett's test (*p* < 0.001), and the correlation coefficients between each dimension and the total score ranged from 0.62 to 0.84. The original study determined the dimensional structures of both scales based on KMO values above 0.80 and the theoretical framework. Therefore, this study directly used the original dimensional divisions of the above scales without repeating exploratory factor analysis.

Validity testing was further conducted in this study. As shown in the validity analysis results in Table 3, the KMO value for parental media literacy was 0.945, for young children's electronic device use was 0.958, for young children's learning quality was 0.957, and the overall KMO value was 0.957. All dimensions passed the Bartlett's test of sphericity, indicating good data validity.

**Table 3:** Validity test results: KMO and Bartlett's test.

Variable	KMO measure of sampling adequacy	Bartlett's test of sphericity		
		Approx. chi-square	df	Sig. (*p*)
Parents' media literacy	0.945	4684.639	171	0.000
Young children's use of electronic products	0.958	6006.826	231	0.000
Young children's learning qualities	0.957	6547.57	253	0.000
Total (All items combined)	0.957	19184.085	2016	0.000

### 2.4 Research procedures and data processing

This study distributed the questionnaire online via Wenjuanxing (a professional online survey platform in China), which was completed by preschoolers' parents. To ensure data validity, the research purpose was explained to parents prior to the survey, and they were requested to provide truthful responses. For data processing and analysis, SPSS 25.0 software was utilized, covering the following specific steps: Conducting a Common Method Bias (CMB) test to assess whether systematic errors existed in the data (see Table 4); Performing descriptive analysis of the basic characteristics of parents' media literacy, preschoolers' use of electronic devices, and preschoolers' learning qualities to understand sample features; Exploring the correlational relationships among the three variables; Employing regression analysis methods to systematically examine the predictive effects and influence magnitudes between the three variables; Further investigating the mediating effect of preschoolers' use of electronic devices (as a mediating variable) between parents' media literacy and preschoolers' learning qualities.

**Table 4:** Common method bias test: results of Harman's single-factor test.

Component	Initial eigenvalues		
	Total	% of Variance	Cumulative %
1	26.18	39.906	39.906
2	7.818	13.216	53.122
3	3.058	4.779	57.9
4	2.019	3.155	61.055
5	1.478	2.31	63.3 65
6	1.333	2.083	65.448
7	1.328	2.075	67.523
8	1.092	1.706	69.229
9	1.038	1.621	70.85

## 3 Research results

### 3.1 Assessment results of common method bias

Common Method Bias (CMB) arises when the measurement subject, environment, and item characteristics are consistent, which tends to lead to spurious correlations between predictor variables and criterion variables. This error causes the distribution of observed data to deviate from the normality assumption, manifesting intuitively as an unreasonable clustering pattern of data in specific score ranges and further contradicting the normality assumption. In this study, the questionnaire data were completed by preschoolers' parents. The single respondent source is prone to causing CMB, which may affect the accuracy of the research results. To reduce the interference caused by CMB and enhance the rigor of the study, Harman's single-factor test was adopted. The test identified 9 factors with eigenvalues >1, and the variance explained by the first factor was 39.906%, which did not reach the critical value of 40%. This indicates that there is no significant CMB in this study.

### 3.2 Overall levels and characteristics of core variables

To understand the basic status of parents' media literacy, preschoolers' learning qualities, and preschoolers' use of electronic devices in the survey sample, and to lay a foundation for subsequent in-depth analysis, this study employed descriptive statistical analysis. Specifically, means and medians were compared to comprehensively grasp the overall levels and characteristics of the three core variables.

#### 3.2.1 Levels and structural characteristics of parents' media literacy

Parental media literacy is divided into six dimensions, which cover the general media literacy requirements for citizens as well as the special needs of family education contexts. In the basic media literacy dimension, high scores indicate that parents can use media efficiently, autonomously, and critically; low scores may reflect passive information reception, insufficient ability to evaluate information, or low media engagement. In the family education media literacy dimension, high scores represent that parents hold scientific perceptions of media education, employ effective management strategies, and maintain positive parent-child interactions; low scores may suggest weak educational awareness, simplistic methods, or insufficient interaction.

After data collection, the obtained data were processed and analyzed. The scale used a 5-point Likert scoring system, where higher scores indicate higher levels of parental media literacy. The scores are presented in the following table:

As shown in Table 5, the average score of parental media literacy was 2.257, indicating a generally low overall level and widespread deficiencies among parents. Among the six dimensions, media information participation had the highest score of 2.733, whereas parent-child media interaction competence had the lowest score at merely 2.176.

**Table 5:** Descriptive statistics of parental media literacy dimensions (*N* = 331).

Dimension	Min	Max	M	SD
Media information exposure	1.000	5.000	2.262	1.134
Media information analysis	1.000	5.000	2.188	0.980
Media participation	1.000	5.000	2.733	1.246
Media education beliefs	1.000	5.000	2.187	0.922
Media education strategies	1.000	5.000	2.201	0.968
Parent-child interaction in media contexts	1.000	5.000	2.176	0.982
Overall parental media literacy	1.000	5.000	2.257	0.843

**Figure 2:**
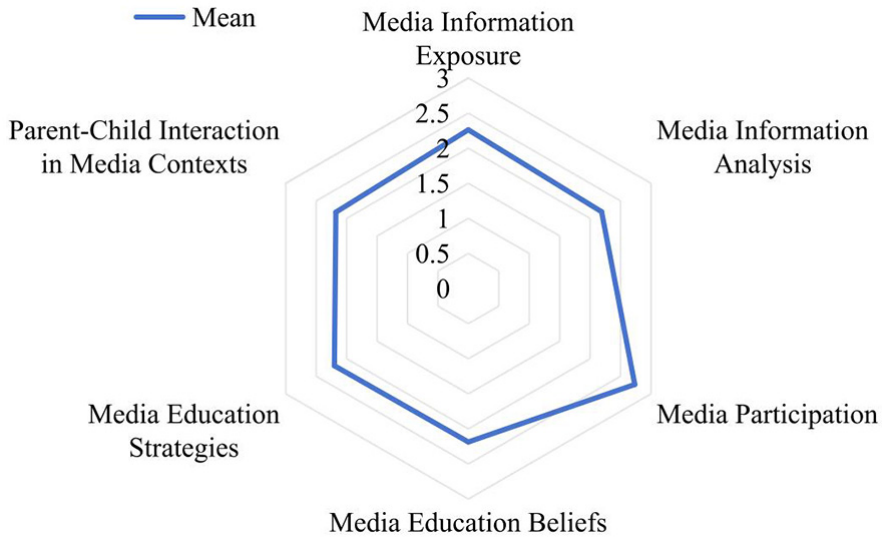
Radar chart of mean scores for parental media literacy dimensions.

To more intuitively reveal the internal structural profile of parental media literacy, a radar chart was plotted based on the mean scores of each dimension, as shown in Figure 2. The results show that all six dimensions fell below the theoretical midpoint of 3, and the overall shape contracted inward, clearly indicating room for improvement across all aspects of parental media literacy in the sample. Further observation of the radar chart reveals a relatively outward bulge in the dimensions of media information participation and media information access, but a noticeable inward indentation in media-related parent-child interaction ability and media education beliefs. This suggests that although parents, as general media users, demonstrate adequate basic abilities in information access and participation, their practical ability to use media for parent-child interaction and family education—as educators of their children—is notably weak.

#### 3.2.2 Overall status of preschoolers' use of electronic devices

**Figure 3:**
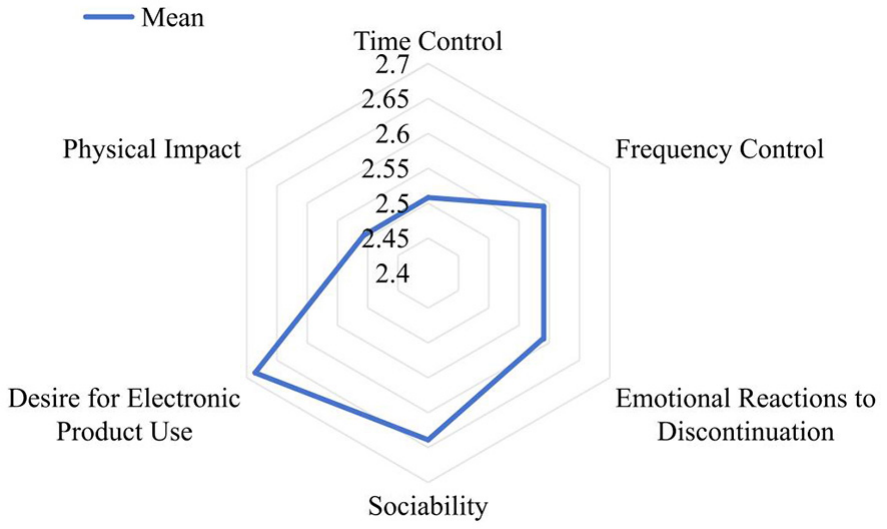
Radar chart of mean scores for young children's use of electronic products dimensions.

Young children's electronic device use behavior is divided into six dimensions, which systematically examine children's dependence on electronic devices and their potential physical and psychological impacts. After data collection, the obtained data were processed and analyzed, and the results of the six dimensions are presented in the following table:

By observing the table above, it can be seen that the mean score for preschoolers' electronic device use in the survey is 2.584, which is below the midpoint (3). This indicates that the electronic device usage of the 331 surveyed preschoolers is suboptimal. Among the six dimensions, the lowest score is observed in physical impacts (2.507), followed by time control with a mean score of 2.508. To further reveal the scores of each dimension of preschoolers' electronic device use, this study plotted a radar chart based on the mean scores in Table 6. Figure 3 presents a distinct fan-shaped structure: the dimension representing desire to use electronic devices forms the protruding part of the chart, while the dimensions related to self-control (time control and frequency control) and health impacts (physical impacts) are significantly concave, forming obvious shortcomings. This structure indicates that preschoolers generally have a strong interest in electronic devices; however, their self-control abilities are weak, and potential negative impacts on their physical health have initially emerged. This suggests that families and educators should not only focus on the content of electronic device use when guiding preschoolers but also strive to address the significant deficiencies in the dimensions of time control and physical health impacts.

**Table 6:** Descriptive statistics of young children's use of electronic products (*N* = 331).

Dimension	Min	Max	M	SD
Time control	1.000	5.000	2.508	0.934
Frequency control	1.000	5.000	2.591	1.126
Emotional reactions to discontinuation	1.000	5.000	2.589	1.119
Sociability	1.000	5.000	2.639	1.084
Desire for electronic product use	1.000	5.000	2.686	1.142
Physical impact	1.000	5.000	2.507	1.112
Overall use of electronic products	1.000	4.818	2.584	0.930

Scores for young children's electronic device use have been reverse-coded. Higher scores indicate more appropriate use of electronic devices by children.

#### 3.2.3 Overall performance of preschoolers' learning quality

Based on Yan Chaoyun's theoretical framework, preschoolers' learning quality is divided into five dimensions: curiosity and interest, initiative, persistence and attention, reflection and explanation, and creativity and invention. As an important foundation for preschoolers' cognitive abilities, social development, and lifelong learning competencies, learning quality directly affects their learning effectiveness and problem-solving abilities. After data collection, descriptive statistical analysis was conducted on the mean scores and standard deviations of each dimension of preschoolers' learning quality, with the specific analysis results presented in Table 7.

**Table 7:** Descriptive statistics of young children's learning qualities (*N* = 331).

Dimension	Min	Max	M	SD
Curiosity and interest	1.000	5.000	2.226	1.028
Initiative	1.000	5.000	2.273	0.932
Persistence and attention	1.000	5.000	2.218	0.906
Reflection and explanation	1.000	5.000	2.222	0.935
Creativity and innovation	1.000	5.000	2.213	0.901
Overall learning qualities	1.000	5.000	2.231	0.793

As shown in the chart, the overall mean score of preschoolers' learning quality is 2.231, which is below the midpoint (3), indicating an overall relatively low level. This suggests that the overall learning quality of the 331 surveyed preschoolers in the study is relatively low. Specifically, preschoolers have the highest mean score in the “initiative” dimension (2.273), while the lowest mean score is observed in the “creativity and invention” dimension (2.213).

**Figure 4:**
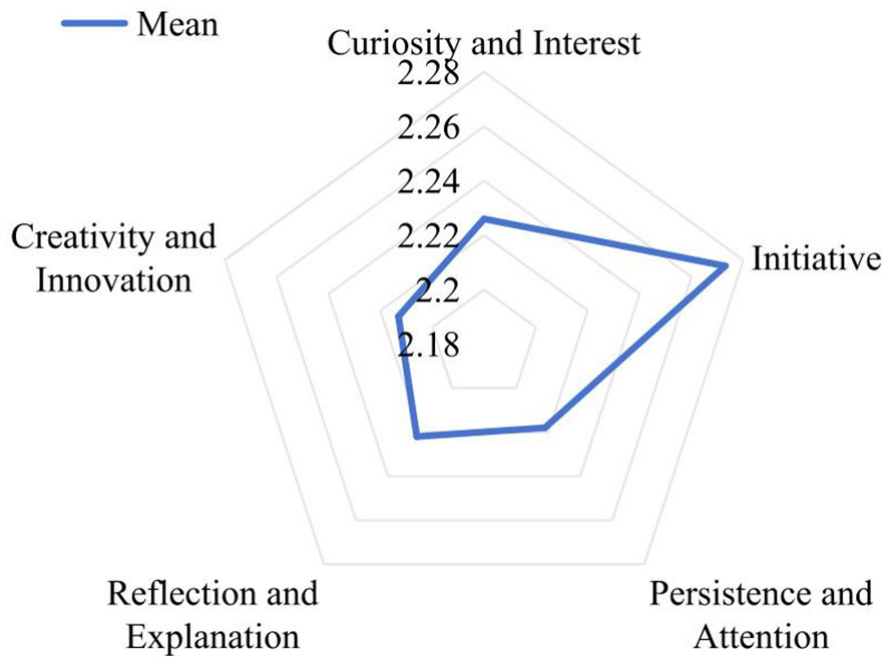
Radar chart of mean scores for young children's learning qualities dimensions.

To obtain an overall picture of young children's learning quality development, this study generated a radar chart based on the mean scores of each dimension, as shown in Figure 4. Specifically, the dimensions of initiative (2.28) and curiosity and interest (2.26) form the two prominent peaks in the chart, representing relatively developed strengths in children's current learning quality. In contrast, the dimensions of creativity and invention (2.18) and persistence and attention (2.22) are noticeably sunken, revealing clear developmental weaknesses. The radar chart indicates that young children currently tend to show strong willingness for spontaneous exploration (high initiative and curiosity), but lack sufficient motivation in persistence, which requires sustained effort and overcoming difficulties, and in creativity, which demands divergent thinking and novel outcomes. This suggests that families and preschool educators should, while protecting children's enthusiasm for exploration, urgently design challenging tasks and open-ended activities to promote a more comprehensive and balanced development of their learning quality structure.

### 3.3 Correlation among parents' media literacy, preschoolers' electronic device use, and preschoolers' learning quality

Correlation analysis is used to explore the relationship and its closeness between variables. The correlation coefficient is employed to measure the degree of the correlation: When the absolute value of the coefficient exceeds 0.7, the two variables exhibit a very strong correlation, indicating an extremely close relationship between them; When the absolute value exceeds 0.4, the correlation is relatively strong; The strength of the correlation is determined by the absolute value of the coefficient: when it is <0.2, the correlation is relatively weak; When the absolute value is <0.1, there is no significant correlation between the two variables. The sign of the correlation coefficient determines the direction of the correlation: a positive sign indicates a positive correlation, while a negative sign indicates a negative correlation.

This study employs Pearson correlation analysis to preliminarily explore the relationships between parents' media literacy and preschoolers' learning quality, between parents' media literacy and preschoolers' electronic device use, and between preschoolers' electronic device use and their learning quality, thereby providing a basis for subsequent regression analysis.

As presented in Table 8 of the correlation analysis: (1) The correlation coefficient between parents' media literacy and preschoolers' electronic device use is 0.473 (*p* < 0.01), indicating a significantly positive correlation between the two; (2) There is a strong positive correlation between parents' media literacy and preschoolers' learning quality, with a correlation coefficient of 0.755 (*p* < 0.01); (3) The correlation coefficient between preschoolers' electronic device use and their learning quality is 0.545 (*p* < 0.01), suggesting a positive correlation between these two variables.

**Table 8:** Results of correlation analysis among parental media literacy, young children's use of electronic products, and young children's learning qualities.

Variable	1	2	3
Parents' media literacy	1		
Young children's use of electronic products	0.473^**^	1	
Young children's learning qualities	0.755^**^	0.545^**^	1

^**^ indicates *p* < 0.01.

### 3.4 Associations of parents' media literacy with preschoolers' electronic device use and learning quality

Regression analysis is employed to explore the interaction effects between variables, and the results can reflect the predictive function of the independent variable on the dependent variable. For example, the predictive effect of parents' media literacy on preschoolers' learning quality can specifically quantify the degree of influence exerted by parents' media literacy on preschoolers' learning quality. To provide more robust evidence for understanding the relationships between variables, this study takes parents' media literacy as the independent variable, preschoolers' learning quality as the dependent variable, and preschoolers' electronic device use as the mediating variable. Before conducting the mediation effect test, linear regression analysis is performed to examine the predictive effects among these three variables.

As shown in Table 9, Parents' media literacy was positively associated with preschoolers' electronic device use (β = 0.522, *p* < 0.01), indicating that for each 1-unit increase in parents' media literacy, preschoolers' electronic device use increases by 0.522 units. In other words, the higher parents' media literacy was related to better preschoolers' electronic device use becomes; Parents' media literacy (β = 0.746, *p* < 0.01) was also positively associated with preschoolers' learning quality: a 1-unit increase in parents' media literacy leads to a 0.746-unit improvement in preschoolers' learning quality, demonstrating that higher parental media literacy is associated with better preschoolers' learning quality; After adding the mediating variable (preschoolers' electronic device use), both parents' media literacy (β = 0.666, *p* < 0.01) and preschoolers' electronic device use (β = 0.154, *p* < 0.01) showed positive correlations with preschoolers' learning quality. Specifically, parents' media literacy was related to preschoolers' electronic device use—higher literacy correlates with better electronic device use quality—which in turn promotes the improvement of preschoolers' learning quality.

**Table 9:** Regression analysis.

Variable		coeff	se	*t*	*p*	*F*
Young children's use of electronic products	constant	1.240	0.196	6.327	0.000	32.611^**^
	Parents' media literacy	0.522	0.054	9.733	0.000	
Young children's learning qualities	constant	0.501	0.115	4.365	0.000	191.759^**^
	Parents' media literacy	0.746	0.031	23.797	0.000	
Young children's learning qualities	constant	0.310	0.117	2.639	0.009	160.059^**^
	Parents' media literacy	0.666	0.034	19.354	0.000	
	Young children's use of electronic products	0.154	0.031	4.917	0.000	

^**^p < 0.05, ^***^p < 0.01.

### 3.5 Test of the mediation effect of preschoolers' electronic device use

Table 10 indicates that the total effect of parental media literacy on young children's learning quality was 0.746. After controlling for the mediating variable (young children's electronic product use), the direct effect value between the two variables was 0.666. The indirect effect referred to the impact of parental media literacy mediated by young children's electronic product use, with an effect value of 0.080. The 95% confidence interval of the total effect ranged from 0.685 to 0.808, while that of the direct effect was 0.598 to 0.734. The confidence interval of the indirect effect through young children's electronic product use was 0.040 to 0.131. None of the three confidence intervals included zero, verifying that young children's electronic product use exerted a significant mediating effect between parental media literacy and young children's learning quality. In the relationship between parental media literacy and young children's learning quality, the indirect effect value of electronic product use was 0.080. Although this effect size was relatively small, it did not weaken the statistical significance of the mediating effect. The significance of mediation analysis is judged by the confidence interval. The findings further proved that electronic product use was only one of the influencing paths, and it played a partial mediating role with an indirect effect of 0.080.

**Table 10:** Mediation effect analysis.

Effect type	Effect	se	LLCI	ULCI
Total effect	0.746	0.031	0.685	0.808
Direct effect	0.666	0.034	0.598	0.734
Indirect effect	0.080	0.023	0.040	0.131

## 4 Discussion

### 4.1 Current status of parental media literacy and its association with young children's electronic device use

For citizens living in a technology-embedded society, the use of electronic products has become a daily habit. Media literacy is related to the quality of parents' own media use and is also associated with their media education for children. However, the results of this study show that the overall level of parental media literacy is relatively low. Descriptive statistics indicate that the mean score of parental media literacy was 2.257, with mean scores of all six dimensions below the midpoint value of 3, suggesting widespread deficiencies in parental media literacy. This finding is consistent with most domestic and international studies. For example, Li and Liu (2021) argued that the vast majority of parents have low awareness of media literacy education. Moon and Shin (2025)) surveyed 514 mothers in South Korea and found that even though children were exposed to digital devices at an early age, mothers still needed external support to guide children in using electronic devices appropriately, indicating that insufficient parental media literacy may be a cross-cultural common phenomenon. Nevertheless, in the dimension of basic media literacy, parents achieved relatively higher mean scores in media information participation and media information access compared with other dimensions. This suggests that parents of young children can proficiently use electronic products to obtain information and engage in social interaction, which can be attributed to the popularity of digital devices and their user-friendly interfaces that lower the barrier to use. In contrast, results in the dimension of family education media literacy differ from previous research. Parents in this survey obtained low mean scores in media-related parent-child interaction ability and media education beliefs, revealing a lack of quality media interaction between parents and young children as well as inadequate media education concepts among parents. However, Ma (2023)) noted that many parents communicate and discuss with their children when the latter use digital media, and recognize that media-based learning is more effective than traditional learning methods. Possible reasons for this discrepancy are as follows: First, sample characteristics differ. In this study, only 15.4% of parents held a postgraduate degree or above, and most families lacked sufficient understanding of media literacy education. Second, parents held biased perceptions of the functions of electronic products, tending to regard them as “calming tools” rather than “educational and interactive carriers.” For instance, they often use short videos or cartoons to quickly distract children in exchange for personal time, instead of co-using or discussing programs with children to understand their thinking. Parental guidance on children's media use remains largely one-way, with many parents believing that paper-based learning materials and outdoor activities are superior to digital learning.

Further data analysis revealed a significant positive correlation between parental media literacy and young children's electronic product use. It indicates that the more reasonably parents guide their children's access to electronic products, the better children's electronic usage habits are. The Convention on the Rights of the Child stipulates that children have the right to freedom of expression and the right to independently select media to explore, obtain and disseminate information. Therefore, young children's use of electronic products should be fully respected. In the digital era, people's ability to use electronic products is closely related to their social adaptation. If parents completely prohibit young children from using electronic products, it will not only restrict their growth, but also cause them to fall behind social development. In Amusing Ourselves to Death, Postman (2015)) warned that television undermines the rational discourse foundation of school education by controlling people's time, attention and cognitive patterns. Parental media literacy is of great significance and helps young children master basic media knowledge and skills. The level of parental media literacy is closely related to the rational use of electronic products by young children, which affects parents' media interaction with their children and educational guidance through media content. However, due to the cross-sectional design of this study, the causal relationship between variables cannot be determined. It is uncertain whether excellent parental media literacy optimizes children's electronic product use habits, or other unmeasured factors such as the overall family educational environment affect both. Future studies can adopt a longitudinal research design to clarify the causal sequence between variables.

### 4.2 Current status of young children's electronic device use and its association with learning quality

Data analysis shows that the average score of children's exposure to electronic devices in this survey was 2.584, which was significantly below the midpoint value. Among the six dimensions, children's desire to use electronic devices scored the highest, while physical impacts scored the lowest, followed by time control. This reflects two prominent problems in children's use of electronic products: negative effects on children's physical health and poor management of usage duration. These results are consistent with the study by Fan (2021)), which indicated that prolonged use of media devices at home first impairs children's physical condition, especially eyesight. Young children show strong interest in electronic devices, maintain frequent exposure to them, and have a high degree of acceptance. A study by Yadav and Chakraborty (2025)) found that the average attention span of 4-year-old children is only about 3 min, and that of 10-year-olds increases to merely 9 min. Therefore, screen time beyond children's physiological attention capacity not only leads to unconscious passive viewing but also increases the burden on their vision and physical health. The trend of younger children using electronic devices has become increasingly evident, with related problems growing more serious. Based on these issues, this study analyzes them from two perspectives: First, children's own developmental characteristics. Children aged 3–6 experience rapid physical growth, and the functions of various bodily systems are still developing, resulting in limited self-control. Their attention is dominated by involuntary attention, while voluntary attention is gradually developing. Consequently, they are easily absorbed in electronic devices with vivid images, rich colors and diverse functions, leading to poor time management and subsequent physical health problems. Second, inadequate parental supervision. Some parents adopt inappropriate parenting styles by using electronic devices as “electronic babysitters” to gain personal time, ignoring the principle of moderation. Even when usage rules are established, implementation is inconsistent, and most parents give in when children cry. In addition, parents lack sufficient understanding of the relationship between screen time, visual development and sleep quality, holding the misconception that “moderate use is harmless.”

Marshall McLuhan pointed out in his media theory that electronic products exert influences on people in various aspects. This study found that the degree of young children's exposure to electronic products was significantly correlated with the development of their learning quality, and excessive use of electronic devices hinders children's healthy growth. This result is consistent with the findings of Zu et al. (2026)). Different types of screen content are differentially associated with early childhood development. Specifically, entertainment-oriented content is negatively correlated with young children's early literacy skills, whereas educational content presents a weak positive predictive effect. Fan (2021)) argued that although electronic product use can provide sensory stimulation and virtual interaction scenarios, the experience obtained in virtual environments is far less authentic than that from real daily life. The communication modes formed by electronic products cannot replace the cultivation of concrete cognition, emotional connections and moral judgment abilities shaped by real social interaction. The findings indicate that educators and parents should pay attention to the scientific management of young children's electronic product use, so as to comprehensively promote the improvement of their learning quality. Moderate electronic product use is associated with children's learning quality. However, due to their physical and mental developmental characteristics, young children have immature willpower and self-control. Without proper supervision, they are highly likely to become addicted to electronic products. Accordingly, parents shall conduct scientific and standardized supervision and guidance on young children's use of electronic products. It should be noted that convenience sampling was adopted in this study, and all participants were from Yunnan Province, China. Yunnan is highly representative of western China in economic development, geographical location and cultural diversity. Nevertheless, the generalizability of the research conclusions to other regions such as China's eastern coastal provinces remains to be further tested in future studies.

### 4.3 Current status of young children's learning quality and the direct and indirect associations of parental media literacy

The survey results show that the average score of young children's learning quality was below the midpoint value of 3, indicating an overall low level. The relatively high score in the dimension of initiative can be attributed to the emphasis on “child-centeredness” in modern preschool education, which encourages children to choose activities independently (such as zone-based play), leading to better performance in active exploration. The dimension of creativity and invention scored the lowest. This may be due to standardized curricula (e.g., templated handicrafts) that restrict children's creative thinking, or the one-way information transmission of electronic products that inhibits children's active creation. Compared with other dimensions of learning quality, creativity and invention impose higher requirements on young children. Although children are naturally interested in novel things, repetitive or unchallenging activities (such as mechanical drills) may make it difficult to sustain their interest. The low level of persistence and attention observed in this study is related to children's own developmental characteristics: their attention stability is poor, they are easily distracted by irrelevant factors, and their attention span is limited. Thus, the low score in persistence and attention is understandable. However, it may also result from the habit of instant feedback from electronic devices—for example, the rapid switching of short videos has reduced children's patience for long-term tasks.

This study verified a significant positive correlation between parental media literacy and young children's learning quality. The results indicate that a higher level of parental media literacy can effectively promote the cultivation of young children's learning quality, confirming a positive correlation between the two variables. This conclusion is consistent with the research findings (Zhou et al., 2024). During children's growth and learning, parents' media use behaviors and attitudes play a leading and exemplary role for their children. A study by Zeng (2021)) noted that parents who focus on improving their children's media literacy not only use the entertainment functions of media but also explore their educational potential, emphasizing the long-term positive impacts of media literacy on children's development. In contrast, families where caregivers have limited media literacy struggle to filter and evaluate media information, resulting in children viewing media merely as a form of entertainment. In the face of complex and diverse media information, parents with strong media literacy can maintain clear judgment amid the flood of information, set good examples for media use, and better fulfill their family education responsibilities. Parents with higher media literacy can quickly identify age-appropriate information for young children, whereas those with lower media literacy may fail to distinguish inappropriate content, exposing children to low-quality materials and hindering the development of their learning qualities. In addition, parents with higher media literacy guide children to acquire knowledge from various media based on their age and interests, broaden their cognitive horizons, stimulate their curiosity for exploration, and lay a solid foundation for improving learning qualities. Therefore, the improvement of young children's learning qualities relies on the enhancement of parents' own media literacy, and fostering a positive family media environment is essential to promoting children's all-round development.

In addition, all data in this study were collected via parental self-reports, which may result in social desirability bias. Although common method bias was examined and controlled, such bias cannot be fully eliminated. This study verified the mediating effect of young children's electronic product use between parental media literacy and young children's learning quality. Specifically, children's electronic product use functioned as a partial mediating pathway in the empirical model. On the one hand, parental media literacy was directly associated with young children's learning quality. On the other hand, parental media literacy also exerted an indirect effect on learning quality by regulating children's exposure to electronic products. Nevertheless, the indirect effect value was only 0.080, indicating a small effect size. This finding suggests that although young children's electronic product use serves as one of the pathways through which parental media literacy is associated with learning quality, its practical contribution is limited. The influence of parental media literacy may operate mainly through other mechanisms, such as parent-child interaction quality and the family learning environment. According to Bronfenbrenner's ecological systems theory, individual development stems from dynamic interactions between individuals and their internal and external environments, highlighting the complex connections between individuals and multi-layered environmental systems. As a key microsystem, the family environment plays a dominant role in early childhood development. Environmental inputs provide critical driving forces for children's developmental trajectories. Parental media literacy is closely linked to the development of children's learning quality, which further confirms its core significance in early childhood upbringing and education. Parents with high media literacy can demonstrate sound educational concepts in daily interactions and fully understand their children's needs. Such interactive patterns promote positive parent-child relationships, thereby generating comprehensive positive effects on children's development, and helping them foster creative thinking and healthy emotional regulation.

## 5 Conclusions and recommendations

Close correlations exist among parental media literacy, young children's electronic product use and their learning quality. Through quantitative analysis, this study explored the complex interactions between the three core variables. Based on the research findings, targeted recommendations were proposed to improve parental media literacy and thereby promote the development of young children's learning quality.

### 5.1 Research conclusions

**Figure 5:**
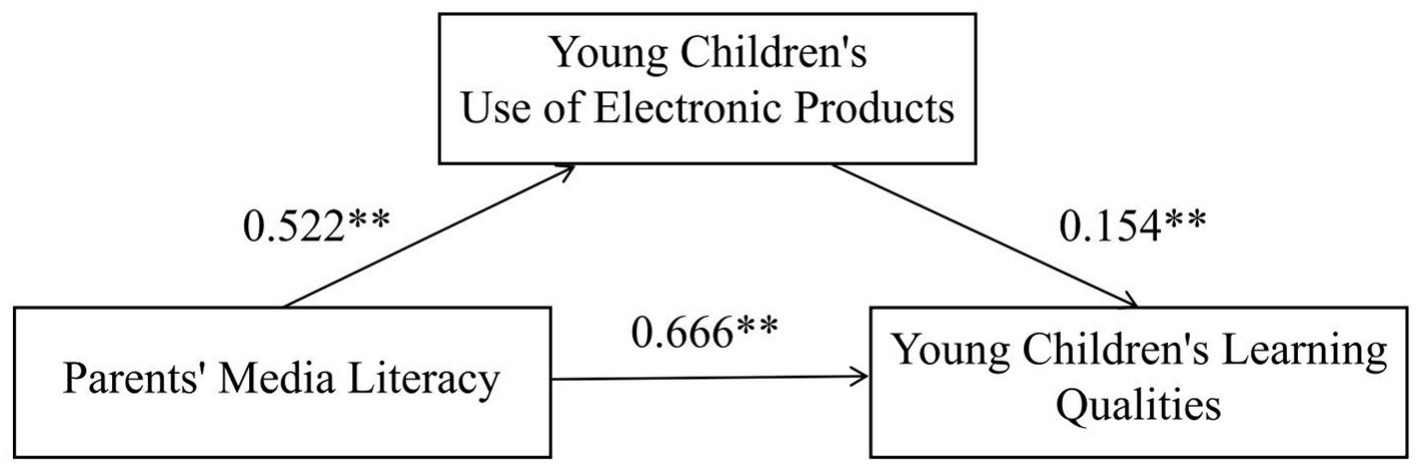
Path diagram of the mediating model with coefficients.

1) There were significant pairwise correlations among parental media literacy, young children's learning quality and electronic product use. Parental media literacy was significantly positively correlated with both young children's electronic product use and their learning quality.

2) Young children's electronic product use played a partial mediating role between parental media literacy and young children's learning quality with a small effect size, and the indirect effect value was 0.080. Parental media literacy is directly associated with young children's learning quality, and is also associated with it through the mediating variable of children's electronic product use (see Figure 5).

### 5.2 Research limitations and prospects

Despite the preliminary findings, this study has several limitations that need to be addressed in future research. First, this study adopted the convenient sampling method with a relatively small sample size, mainly focusing on families in Yunnan Province, which may affect the external validity of the conclusions. Future studies could use stratified sampling to expand the sample size and cover more regions and family types. Second, regarding the measurement instruments, the scales used in this study were adopted from items and dimensional structures of previous research. In future studies, questionnaire items could be designed more rigorously and carefully to achieve more objective and scientific research. Third, in terms of the research model, young children's electronic device use only played a partial mediating role, indicating that there are other pathways through which parental media literacy is associated with children's learning quality. Future studies could introduce more variables (such as the quality of parent-child interaction, parenting style, etc.) to explore chain mediation or moderated mediation models, and adopt a longitudinal follow-up design to clarify causal directions.

### 5.3 Educational recommendations

#### 5.3.1 Improve parents' media literacy and foster a positive family media environment

With the development of society, basic media literacy has gradually become an important cornerstone for citizens to stand firm in the new media era. The improvement of parents' media literacy is not only a survival skill to keep pace with the trend of the times but also provides solid support for the cultivation of preschoolers' media literacy through parents' media-related initiatives. As the primary setting for preschoolers' exposure to electronic devices, the family environment is where parents' media literacy exerts an influence on preschoolers, thereby affecting their media literacy level. Research has shown that parents' media literacy is not only significantly associated with preschoolers' learning quality but also serves as a key factor related to it.

First, parents are advised to establish a scientific concept of media use. The information age emphasizes digital parenting—parents must not only correctly recognize the instrumental attribute of electronic devices, avoiding extreme attitudes of excessive dependence or complete rejection, but also attach importance to the value of family media education. Most of preschoolers' exposure to electronic devices occurs at home, and as their first teachers, parents can leverage the characteristics of family education (being targeted and integrating family affection) to cultivate their children's media literacy. As a symbol of modern technology, electronic devices are related to preschoolers' growth in both positive and negative ways. Appropriate application can not only serve as an important means to promote preschoolers' cognitive development, broaden their horizons, and enhance their learning motivation but also deepen their personalized learning process and foster emotional development under proper guidance. Well-designed interactive materials and educational software can create a safe and enlightening learning environment for children, effectively promoting their balanced development in knowledge accumulation, ability enhancement, and sociality. According to current research findings, most parents generally believe that the adverse impacts of exposing preschoolers to electronic devices too early far outweigh the potential benefits. Therefore, to promote healthy family interactions and children's all-round development, parents may need to adhere to appropriate concepts and adopt scientific strategies to improve their comprehensive media literacy.

Secondly, parents could broaden their media-related knowledge and skills and master rational media education strategies. Based on preschoolers' age characteristics and developmental levels, parents may select appropriate, positive, and age-appropriate content from their children's perspective. They could learn to use professional media content evaluation tools or platforms to check reviews and recommendations of relevant content, thereby guarding the quality of media content for preschoolers. The family's exemplary role has a lasting impact, yet many parents fail to recognize their own value: they prohibit their children from using electronic devices while becoming addicted to them themselves. In such cases, attempts to regulate and guide preschoolers' use of electronic devices may be counterproductive. Parents themselves may set an example of healthy electronic device use, reducing behaviors such as mindlessly scrolling through mobile phones or indulging in games in front of their children. Meanwhile, they could establish a communication mechanism with preschoolers regarding electronic device use, fully listening to their children's thoughts and needs, and exchanging feelings and experiences related to usage.

#### 5.3.2 Cultivate preschoolers' habits of using electronic devices and give play to their positive roles

Control preschoolers' electronic device usage time and improve the quality of such use. Based on preschoolers' age characteristics, parents may carefully allocate and disperse usage time to avoid prolonged continuous use. For example, limit each session to 5–10 min, followed by rest periods such as doing eye exercises or physical activities to protect preschoolers' visual and physical health. Given the vast amount of information online and preschoolers' weak ability to distinguish right from wrong, parents could screen content oriented to preschoolers' learning and developmental needs, selecting electronic device content that is highly educational and engaging.

Formulate rules for electronic device use and strengthen the supervision and control of preschoolers' usage. Parental supervision and guidance are crucial for cultivating preschoolers' healthy electronic device use habits. Parents may enhance pre-use management by scientifically screening content, emphasize companionship during use, and adopt appropriate intervention measures (e.g., parent-child co-use) to improve the quality of preschoolers' electronic device use.

#### 5.3.3 Attach importance to the cultivation of preschoolers' learning quality and lay a foundation for their lifelong development

Parents may adopt a correct attitude toward electronic devices, regarding them as daily life tools rather than necessities, and pass on this concept to preschoolers. In daily life, parents may spend their leisure time participating in activities with preschoolers, exposing them to new things and the natural environment. When preschoolers show strong curiosity, parents could seize the educational opportunity to guide them through a complete inquiry process through methods such as jointly searching for information, on-site observation, or simple experiments. This helps consolidate preschoolers' initiative advantages, promote the development of their reflective and explanatory abilities, and achieve positive transfer from strengths to weaknesses.

Parents could grasp the laws of preschoolers' physical and psychological development and respect their subject status. By deeply understanding these development laws, parents may strive to build a close parent-child relationship in the practice of guiding preschoolers' exposure to electronic devices, while continuously maintaining and stimulating their learning interest to help them form excellent learning habits and qualities. Create a positive growth ecological environment for preschoolers, allowing them to understand the surrounding world in a favorable cultural atmosphere, broaden their horizons, and accumulate knowledge, thereby continuously promoting the development of their learning quality.

## Data Availability

The data analyzed in this study is subject to the following licenses/restrictions: The dataset will be provided upon reasonable request. Requests to access these datasets should be directed to Guzhenglin@126.com.
