# Supplementary material for: Research on the relationship between parents' media literacy and preschoolers' learning quality: the mediating role of preschoolers' electronic device use
Source: Front Psychol. 2026 Jun 16;17:1792526. doi: 10.3389/fpsyg.2026.1792526 (PMC13317070; doi:10.3389/fpsyg.2026.1792526)
Supplement: Supplementary file 1 [file Supplementary_file_1.pdf]

Yunnan Normal University Faculty of Education Research Ethics Review Application Form

（No.： YNNUJY-2025-001 ）

<table>
<tr><td>Project Name</td><td colspan="8">Research on the Relationship Between Parents' Media Literacy and Preschoolers' Learning Quality: The Mediating Role of Preschoolers' Electronic Device Use</td></tr>
<tr><td>Type of Project for Review</td><td colspan="8">□ Project to be applied for research □ Extended review for approved projects ☑Survey</td></tr>
<tr><td rowspan="2">Project Source (For Approved Projects)</td><td colspan="8">☑ Government □ Domestic enterprises and public institutions □ Foreign & international organizations □ Others____________</td></tr>
<tr><td colspan="8">Name of Funding Agency：Department of Social Sciences, Ministry of Education</td></tr>
<tr><td>Project Level</td><td colspan="8">□ National Major Project □ National Key Project □ General National Project
□ Provincial & Ministerial Major Project □ Provincial & Ministerial Key Project
☑ General Provincial & Ministerial Project
□ Municipal & Departmental Major Project □ Municipal & Departmental Key Project
□ General Municipal & Departmental Project □ Others____________</td></tr>
<tr><td>Project Duration</td><td colspan="8">March 1, 2025 — March 1, 2026</td></tr>
<tr><td rowspan="2">Brief Information of Applicant (Project Principal)</td><td>Name</td><td>Zhenglin Gu</td><td>Affiliation</td><td>Faculty of Education</td><td>Staff ID</td><td>4363</td><td>Contact Number</td><td>15812096183</td></tr>
<tr><td>Professional Title</td><td>Associate Professor</td><td>Academic Degree</td><td>Doctor</td><td colspan="2">Major & Research Field</td><td colspan="2">Preschool Education</td></tr>
<tr><td>Cooperating Institution</td><td colspan="3">Kunming Medical University</td><td colspan="3">Person-in-charge & Contact of Partner</td><td colspan="2">Yang Wang13529304950</td></tr>
<tr><td>Submitted Review Materials</td><td colspan="8">□ Project Application Documents ☑ Survey Plan ☑ Informed Consent Form □ Other</td></tr>
<tr><td colspan="9">Research Significance and Content
This study explores the correlation between parental media literacy and young children's learning quality, as well as the mediating effect of young children's electronic product usage. Online questionnaires are distributed via Wenjuanxing, and data are analyzed with SPSS for descriptive statistics, correlation analysis and regression analysis. The research findings will provide empirical evidence and educational suggestions for improving parental media literacy, regulating children's use of electronic products and boosting young children's learning quality.</td></tr>
<tr><td colspan="9">Main Research Technologies
Online questionnaire (Wenjuanxing), SPSS statistical analysis</td></tr>
<tr><td colspan="9">Undertaking by Applicant
I hereby confirm that all information and attached documents submitted are true and the research contains no content prohibited by the Ethics Committee. If this application is approved, I will abide by relevant ethical laws, regulations and codes of conduct.
Signature of Applicant (Project Principal): Zhenglin Gu
Date: 10 February 2025</td></tr>
</table>

Review Comments

Conclusion：☑Approved　　□Disapproved

Faculty of Education, Yunnan Normal University

云南师范大学 教育学部

Date: 10 February 2025
